# Proposal for computer-aided diagnosis based on ultrasound images of the kidney: is it possible to compare shades of gray among such images?

**DOI:** 10.1590/0100-3984.2019.0138

**Published:** 2021

**Authors:** Gustavo Lopes Gomes de Siqueira, Robson Pequeno de Sousa, Ricardo Alves de Olinda, Carlos Alberto Engelhorn, André Luiz Siqueira da Silva, Juliana Gonçalves Almeida

**Affiliations:** 1 Faculdade de Ciências Médicas de Campina Grande (Unifacisa), Campina Grande, PB, Brazil.; 2 Universidade Estadual da Paraíba (UEPB), Campina Grande, PB, Brazil.; 3 Pontifícia Universidade Católica do Paraná (PUCPR), Curitiba, PR, Brazil.

**Keywords:** Diagnosis, computer-assisted, Kidney/diagnostic imaging, Ultrasonography/methods, Ultrasonography, interventional/methods, Image processing, computer-assisted/methods, Diagnóstico por computador, Rim/diagnóstico por imagem, Ultrassonografia/métodos, Ultrassonografia de intervenção/métodos, Processamento de imagem assistida por computador/métodos

## Abstract

**Objective:**

To compare ultrasound images of the kidney obtained, randomly or in a controlled manner (standardizing the physical aspects of the ultrasound system), by various professionals and with different devices.

**Materials and Methods:**

We evaluated a total of 919 images of kidneys, obtained by five professionals using two types of ultrasound systems, in 24 patients. The images were categorized into four types, by how they were acquired and processed. We compared the gray-scale median and different gray-scale ranges representative of virtual histological tissues.

**Results:**

There were statistically significant differences among the five professionals, regardless of the type of ultrasound system employed, in terms of the gray-scale medians for the images obtained (*p* < 2.2e-16). Analyzing the four categories of images-a totally random image (without any standardization); a standardized image (with fixed values for gain, time gain control, and dynamic range); a normalized version of the random image; and a normalized version of the standardized image-we determined that the random image, even after normalization, differed quite significantly among the professionals (*p* = 0.006098). The analysis of the normalized version of the standardized image did not differ significantly among the professionals (*p* = 0.7319).

**Conclusion:**

Our findings indicate that a gray-scale analysis of ultrasound images of the kidney performs better when the image acquisition process is standardized and the images undergo a process of normalization.

## Abstract (pt)

**Objetivo:**

Comparar imagens renais ultrassonográficas obtidas de maneira aleatória e controlada (padronizando fatores físicos do aparelho de ultrassom) por diferentes profissionais e aparelhos.

**Materiais e Métodos:**

Foram obtidos quatro tipos de imagens, de acordo com sua aquisição e processamento por cinco profissionais e dois tipos de aparelhos de ultrassonografia, em 24 pacientes, totalizando 919 imagens. Comparamos a mediana de escala de cinza e diferentes intervalos de tons de cinza representantes de tecidos histológicos virtuais.

**Resultados:**

As medianas de escala de cinza de imagens renais obtidas por dois tipos de aparelhos foram estatisticamente diferentes (*p* < 2.2e-16). Analisando os quatro tipos de imagens, partindo de uma totalmente aleatória (sem qualquer padronização), uma padronizada (fixado o ganho, *time gain control* e *dynamic range*), e essas duas passando por um processo de normatização, obteve-se que a imagem aleatória é totalmente diversa entre os profissionais (*p* = 0,006098), mesmo passando pelo processo de normatização. A imagem padronizada, após passar pelo processo de normatização, apresentou resultados equivalentes, não possuindo diferença estatística (*p* = 0,7319).

**Conclusão:**

Constatou-se que na análise de tons de cinza deve-se usar um mesmo tipo de máquina e uma imagem em que sejam padronizados aspectos físicos, passando por um processo de normatização/padronização.

## INTRODUCTION

The evaluation of the renal parenchyma remains subjective to the comparison of its echotexture in relation to the liver, without specific parameters in relation to factors such as the percentage of pixels^(1)^.

Human vision can perceive only 16-32 shades of gray. Ultrasound generates up to 256 shades of gray, 16 times more than the human eye can perceive^(2)^. The computerized analysis of shades of gray can reveal subtle changes in a given structure over time, changes that are initially imperceptible to the human visual system but become visible after computerized gray-scale mapping^(3)^, transforming the image into a three-dimensional digital file, brightness being the third dimension^(4)^.

**Figure 1:**
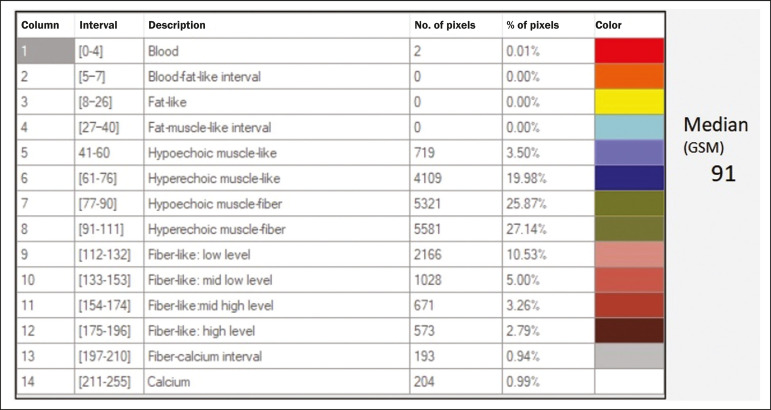
Pixel brightness ranges and the probable corresponding tissues on ultrasound.

**Figure 2:**
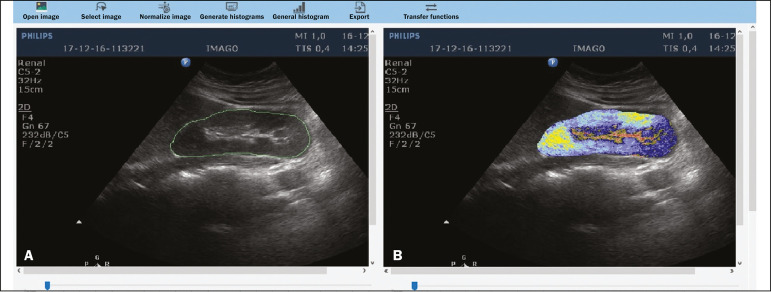
Software (CAD) prototype. Image showing manual segmentation of the renal contour (**A**) and the pseudocolor version of the image (**B**).

Ultrasound tissue characterization is based on two parameters^(5)^: quantification of specific percentages of shades of gray at pre-established ranges (specific brightness ranges; Figure 1); and color mapping of the image (Figure 2), which improves the level of perception by the human visual system. An important part of this evaluation is determination of the gray-scale median (GSM), which marks the division between the pixels with greater and those with lesser brightness in the selected area. The GSM thus divides a sample in half, being different than the mean and not affected by the values to the right or left of it. Because it is not so influenced, the median is more important than the mean in some statistical studies.

The computerized assessment of shades of gray in ultrasound images has been widely used in the evaluation of atherosclerotic carotid plaque^(6,7)^, in which a GSM < 25 indicates a greater risk of stroke. To make the images more uniform and less dependent on tissue attenuation^(4,5,7)^, we resized the gray-scale ranges (Figure 1). The proportional distribution of shades of gray within the selected region of the kidney was studied in 14 brightness ranges, and the GSM was determined. The benefit of this evaluation would be, for example, in the ultrasound monitoring of kidney transplant recipients, in which spectral parameters are used, although potentially only after the parenchyma and its echotexture have changed, as can happen in cases of graft rejection. Given the limits of the human visual system, such changes can go unnoticed without the aid of a computerized system. The gray-scale and GSM ranges have already been described in normal patients by investigators using the parameters previously cited in studies analyzing shades of gray of atherosclerotic carotid plaque^(2,4,7-9)^. In such studies, the image is “standardized” with respect to two points: the adventitia is assigned a pixel value of 200 and the lumen is assigned a pixel value of 0, which effects a linear change in all other values, in order to standardize images obtained by different examiners with different devices. In previous studies evaluating renal conditions, the posterior muscular fascia was assigned a pixel value of 200 and the darkest part of the image was assigned a pixel value of 0^(4,5,10)^. In one of those studies, in which a kidney transplant recipient was evaluated, changes in the GSM and gray-scale ranges facilitated the early identification of acute graft rejection^(10)^.

The main problematic aspect of ultrasound is that the use of different devices by different examiners can generate totally different shades of gray. To compensate for that, we used the “standardization” method mentioned above^(2,4,5,7-9)^, although it must be taken into account that evaluation of the kidney is very different from evaluation of the carotid artery, in which the target has a superficial presentation and is easily visualized. Then leaves us with the question of whether we can evaluate shades of gray in renal images acquired with different devices and by different examiners.

## MATERIALS AND METHODS

Cross-sectional, observational, descriptive study with an analytical component. The study was approved by the Research Ethics Committee of Paraíba State University, in the city of Campina Grande, Brazil (Reference no. 86802617.5.0000.5187; opinion no. 2,954,650), and all participants gave written informed consent.

In collaboration with a computer engineering team, we developed a software prototype using the C++ language. The prototype was created in the Microsoft Visual Studio Community 2015 integrated development environment for Windows (version 14.0). With our software, it is possible to “standardize” an image in an adapted manner^(4,5,10)^, stabilizing the pixels in relation to a single point (although other studies have used two points), which will be the posterior renal fascia. After the use of the zoom command and manual segmentation of the fascia, the operator clicks on the normalization (standardization) command and the GSM of the segmented area automatically changes to 200. The mathematical change occurs not only in the segmented area of the renal fascia but also throughout the image, changing all pixels in the image in accordance with the change in the segmented fascia.

The mathematical change around the pixel variation will occur due to a normalization factor. The normalization factor (*Fn*) is defined as in Eq. 1:

(1) $$\mathrm{Fn}=\left[\left({\mathrm{medF}}_{0}-200\right)/{\mathrm{medF}}_{0}\right]$$

where *medF_0_* is the original GSM of the selected fascia.

After calculating this factor, we used the transfer function defined in Eq. 2 to normalize the image to the GSM of the fascia of the selected region. To apply Eq. 2, the following criteria must be observed:

(2) $$\mathrm{If}\;{\mathrm{medF}}_{0}\leq 200:f\left(r\right)=r\left(1+F_{n}\right)$$

(3) $$\mathrm{If}\;{\mathrm{medF}}_{0}>200:f\left(r\right)=r\left(1-\mathrm{Fn}\right)$$

where *f* is a given pixel and *r* is the pixel intensity level in the image.

The use of image normalization can be exemplified as follows:

If the *medF_0_* is 220, the normalization factor will be calculated as in Eq. 1:

$$\mathrm{Fn}=\left[\left(220-200\right)/220\right]=0.09$$

To normalize the original image, Eq. 3 is used. Assuming that the intensity of a given pixel in the original image is 50, according to Eq. 3 the normalized pixel will assume the following value: $f(50)=50\left(1-0.09\right)=46$.

In addition to making it possible to normalize the image, the software applies the current gold standard for renal segmentation in ultrasound imaging, which continues to be the manual method^(11,12)^. The computer-aided diagnosis (CAD) prototype reads the proportional distribution of pixels within the segmented image and uses 14 brightness ranges to create a pseudocolor image (Figures 1 and 2).

Twenty-four volunteers were included in the study. All of the volunteers underwent ultrasound of the right and left kidney by five specialist sonographers, designated physicians 1 through 5. Physicians 1, 2, and 3 used the same system, a Philips HD11 XE (Philips Healthcare, Eindhoven, The Netherlands) with a 3-7 MHz convex transducer. Physician 4 used a GE Logiq S7 system (GE Healthcare, Chicago, IL, USA) that had been in use for three years, with a 6-9 MHz convex transducer. Physician 5 used another GE Logiq S7 system (GE Healthcare) that had been in use for two years, also with a 6-9 MHz convex transducer. In the renal examination, performed with the volunteer in the lateral position, two methods were employed. The first was designated the random method, in which the usual preset for renal examination was employed and the physician manipulated the gain, the time gain control (TGC), and all other factors. The image thus obtained was designated the sample image. The second method, designated the standardized method, employed a “control” image, in which we created a preset with a dynamic range fixed at 70-80 dB and the gain fixed at 100 dB, not being able to modify the parameters described, stabilizing the vertical TGC in the renal area in order to generate the best possible image^(4,6-10)^. The image thus obtained was designated the control image. After a total of 480 images, 240 of each (sample and control images), had been saved to the ultrasound system, the images were transferred to a computer, with the file extension .bmp, at a resolution of 800 × 600 pixels.

Each sample or control image, of the right or left kidney, was subjected to analysis in the software created, finally producing what we call a random image and a standardized image, according to the following procedure:

- Manual segmentation of the renal contour
- Analysis with acquisition of the GSM and the shades of gray
- Acquisition of the 14 brightness ranges described in Figure 1
- Creation of a pseudocolor version of the selected image (Figure 2).

Subsequently, the random and standardized images were submitted to a procedure for creating the “standardized/normalized” images, as follows:

- Preprocessing with standardization/normalization of images after choosing a fixed point that serves as a reference for the number 200 on the gray scale, using the renal fascia^(4,5,10)^
- Manual segmentation of the renal contour
- Analysis with acquisition of the GSM and the shades of gray in the selected image, together with acquisition of the 14 brightness ranges (Figure 1)
- Creation of a pseudocolor version of the selected image (Figure 2)

The images thus created were called random-normalized (n = 240) and standardized-normalized (n = 240).

Images in which it was difficult to visualize the renal fascia or renal alterations were excluded. A total of 41 images were thus excluded: 20 from a patient who had an extensive staghorn kidney stone in the left kidney; and 21, from three patients, that were not perfect candidates for the standardization procedure. After excluding the cited images, 919 images resulted for the study: 231 random images, 229 standardized images, 230 random-normalized images, and 229 standardized-normalized images.

### Statistical analysis

The data were entered into a Microsoft Excel 2016 spreadsheet. After being organized, the main descriptive statistics were presented. The GSM was calculated and analyzed specifically. Percentages were interpreted as continuous variables. Mean, standard deviation, minimum and maximum values were calculated on the basis of the descriptive statistics analyzed with the statistical software R (The R Foundation, Vienna, Austria).

To assess the adequacy of the proposed statistical modeling to describe the observations, the normality and independence of the variables were verified with the Anderson-Darling normality test. With this procedure, we sought to create the theoretical conditions necessary for performing univariate statistical analyses. To identify differences between physician and imaging factors, we used the nonparametric Kruskal-Wallis test, which is analogous to the analysis of variance (ANOVA) F test. To identify differences between the medians, we then used the Wilcoxon-Mann-Whitney test for independent samples. In all tests, the level of significance was set at 5% (*p* < 0.05), as analyzed with the aid of the software R.

Two scenarios were devised: scenario 1-analysis among the five physicians and the three types of ultrasound systems, evaluating the possibility of comparing images produced by different devices and physicians; scenario 2-comparison among the three physicians who used the same ultrasound system (physicians 1, 2, and 3) and among the four types of images (random, standardized, random-normalized, and standardized-normalized).

## RESULTS

**Figure 3:**
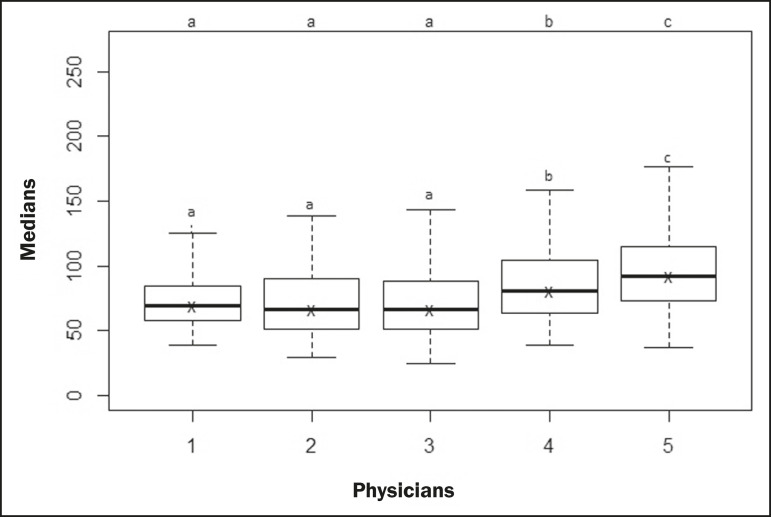
Univariate analysis among the five physicians, using GSM as a variable (*p* < 2.2e-16).

**Scenario 1**-The five types of physicians were compared, in relation to the GSM variable, using the Kruskal-Wallis test (ANOVA), and significant differences were observed (*p* < 2.2e-16). To identify intraindividual differences, the Wilcoxon-Mann-Whitney test for independent samples was applied (Figure 3).

**Scenario 2**-A comparison was made among the physicians who used the same ultrasound system (physicians 1, 2, and 3) and the four different types of images (random, standardized, standardized-normalized, and random-normalized). The GSM and the 14 brightness ranges were used as variables for comparison.

### Random images

For each random image, the Kruskal-Wallis test (ANOVA) was used in order to identify differences among the levels of the physician factor (physicians 1, 2, and 3), the median of the GSM being used as a variable. There was a statistically significant difference among the physicians (*p* = 0.006098). The Wilcoxon-Mann-Whitney test was used in order to compare physicians 1, 2, and 3 in terms of the GSM and of the 14 brightness ranges of the random image, statistical differences being observed between physicians 1 and 2, for 11 brightness ranges and for the GSM, as well as between physicians 1 and 3, for 5 brightness ranges and for the GSM. Notably, there was no statistical difference between physicians 2 and 3.

### Random-normalized images

A random-normalized image is a random image that has undergone the normalization process. For each random-normalized image, the Kruskal-Wallis test (ANOVA) was used in order to identify differences between the levels of the physician factor (physicians 1, 2, and 3), the median of the GSM being used as a variable. The GSM did not differ significantly among the physicians (*p* = 0.08115). The Wilcoxon-Mann-Whitney test was used in order to identify differences among physicians 1, 2, and 3 in terms of the GSM and of the 14 brightness ranges of the random-normalized images, statistical differences being observed between physicians 1 and 2 for four brightness ranges (although not for the GSM), as well as between physicians 1 and 3 for two brightness ranges. There was no statistical difference between physicians 2 and 3.

### Standardized images

Standardized images represent the most widely used type of image acquisition^(4,5,7-10)^. Comparison of the 14 brightness ranges and the GSM is crucial to describing the equivalence of the images. For each standardized image, the Kruskal-Wallis test (ANOVA) was used in order to identify differences between the levels of the physician factor (physicians 1, 2, and 3), the GSM being used as a variable. The GSM did not differ among the physicians (*p* = 0.9472). To identify differences in the GSM and of the 14 brightness ranges of the standardized images, the Wilcoxon-Mann-Whitney test was used, a statistical difference being observed only between physicians 1 and 3 and only in one brightness range.

### Standardized-normalized images

A standardized-normalized image is a standardized image that has undergone the normalization procedure. For each standardized-normalized image, the Kruskal-Wallis test (ANOVA) was used in order to identify differences between the levels of the physician factor (physicians 1, 2, and 3), the GSM being used as a variable. The GSM did not differ among the physicians (*p* = 0.7319). The Wilcoxon-Mann-Whitney test was used in order to determine whether there were any differences between the GSM and of the 14 brightness ranges in the standardized-normalized images generated by physicians 1, 2, and 3. There were no statistical differences among the physicians in terms of the brightness ranges obtained.

## DISCUSSION

Various authors have concluded that the processing and analysis of ultrasound images have become totally dependent on the examiner^(1)^. A study attempting to analyze shades of gray in ultrasound images atherosclerotic plaques produced dissonant results regarding the risk of ischemic stroke^(13)^. The normalization procedure has been shown to partially eliminate the variability among ultrasound images obtained by different professionals^(2,7-9)^. However, all of the studies cited evaluated atherosclerotic plaques in images of the carotid artery, which are obtained relatively easily because of the superficial location of the artery.

There have been multiple studies involving gray-scale analysis of renal ultrasound images, using the same normalization principle, adapted for renal images^(4,5,10)^. However, the main question regarding the evaluation of renal images is whether they can be compared among different professionals and ultrasound systems.

In the present study, four types of images were used, depending on whether they were standardized or normalized. As a result, the GSMs obtained by the physicians who used the same ultrasound system (physicians 1, 2, and 3) differed from those obtained by the physicians who used another type of ultrasound system (physicians 4 and 5). This leads us to suggest that it is not possible to compare images obtained with different ultrasound systems, because the variability is statistically significant. Despite that result, we cannot be sure that such an analysis is precluded, given that only one aspect (variable) was compared. We also have no explanation for our finding that the results differed between physicians 4 and 5, who used ultrasound systems of the same model, although with different serial numbers (difference in use of one year). To our knowledge, there have been no studies comparing normalized images obtained with different ultrasound systems. Our findings suggest that the same ultrasound system should be used in order to improve the comparison and analysis of shades of gray. The question that remains is whether it is possible to compare renal ultrasound images obtained by different professionals with the same ultrasound system.

For the random image and its variant after normalization-the random-normalized image, in which no physical aspect of the image was standardized-the results demonstrated total variability between those obtained by different professionals, making it impossible to use these types of images for comparison.

In the standardized image, there was no significant change in the most important variable (GSM). However, there was a statistically significant, albeit marginal, difference between physicians 1 and 3.

For the standardized-normalized images, there were no statistical differences among physicians 1, 2, and 3, supporting the hypothesis of equivalence of the images and homogenization after the normalization procedure. Standardized-normalized images have been used in most of the major studies describing the analysis of shades of gray^(2,4-6,8-10)^.

We propose that CAD be employed for the analysis of renal images, using images that have been standardized and normalized. Most of the studies of the use of CAD in renal ultrasound imaging have applied it in image segmentation. One of its most rarely used applications, in the context of renal evaluation, is to identify kidney stones^(14)^. A review of the current state of development of new techniques for the computerized analysis of renal ultrasound images showed that there is a paucity of studies on the topic^(15)^. Even after all of the technological advances, renal sonography continues to be totally dependent on the examiner. There is a consensus that the development of CAD applications for renal imaging is too limited and needs to be advanced. Renal elastography might be a future direction for this technology^(16,17)^. Since its invention in 1960 until the beginning of the 21st century, despite all advances, CAD continued to be employed as a “second opinion”, as an aid to the more rapid decision made by the health care professional. Recent research suggests a radical change in the main functions of CAD applications, which are moving from being mere adjuvants to being more complex systems, with the capacity for knowledge and learning, using not only images but also informative data about the condition of the patient, which can result in a more accurate decision-making process^(18)^.

## CONCLUSION

The use of the same ultrasound system to produce all of the images evaluated appears to confer greater credibility on the results. However, because we did not randomize the various types of ultrasound systems and physicians, we cannot rule out the possibility that images produced by different ultrasound systems could be compared. In the procedure for the acquisition of renal images, it is suggested that modifiable physical aspects of the image (e.g., TGC, total gain, and dynamic range) be standardized and that the images be submitted to a post-acquisition normalization procedure. Because there are few data in the literature regarding the computerized analysis of renal ultrasound images, it would be interesting to use a CAD application that focuses on the variations in shades of gray.
